# Fishing for adaptive epistasis using mitonuclear interactions

**DOI:** 10.1371/journal.pgen.1006662

**Published:** 2017-03-31

**Authors:** David M. Rand

**Affiliations:** Department of Ecology and Evolutionary Biology, Brown University, Providence, Rhode Island, United States of America; Fred Hutchinson Cancer Research Center, UNITED STATES

Mitochondria provide us with the energy to get up and do what needs to be done. They do this with 37 genes encoded in their own genome and more than 1,200 genes from the nuclear genome, whose gene products are targeted to mitochondria after being translated on cytosolic ribosomes. This complex intergenomic communication is a hallmark of eukaryotic life and has been under continuous refinement for more than 10^9^ years, following the fusion of eubacterial and archaebacterial cells [1]. How these two genomes cooperate in the face of conflicting modes of genetic transmission, distinct evolutionary pressures of mutation and recombination, and oxidative damage brought on by the very activity of mitochondria, has puzzled and motivated biologists for decades. A central challenge is to understand the coordinated modes of communication that maintain biochemical, physiological, and evolutionary coadaptation of the nuclear and mitochondrial compartments. The traditional view is that central administration (the nucleus) maintains the activities of these hardworking laborers (mitochondria) by sending regulatory agents (nuclear gene products) to organelles spread across the cellular landscape. A growing body of literature points to diverse ways that these energy factories voice their concerns about the government of mitonuclear interactions and send retrograde signals back to the nucleus, altering its control over mitochondria [2]. Studies of these patterns and processes hope to resolve some pretty fundamental questions about life on earth: the causes of aging and disease [3–5], the metabolic bases of adaptation and Darwinian fitness [6], the genetics of speciation [7], the origin of sex and recombination [8, 9], the battle of the sexes, and genomic conflict [10].

Most questions about mitonuclear interactions focus on the oxidative phosphorylation pathway (OXPHOS), the ATP producing process that includes four multisubunit protein complexes of the electron transport chain (ETC) and a fifth complex that phosphorylates ADP to produce ATP. These OXPHOS complexes, embedded in the mitochondrial inner membrane, include all of the 13 proteins encoded in animal mitochondrial DNA (mtDNA) plus ~78 proteins encoded on nuclear chromosomes. Two other classes of mtDNA genes, transfer- and ribosomal- RNAs, are needed to express mtDNA encoded proteins, and their functions require nuclear encoded proteins for transfer RNA (tRNA) charging, ribosome function, and RNA processing. Because these sets of genes involve physical interactions between mtDNA- and nuclear-encoded gene products, they are logical targets for studies of mitonuclear communication, coordination, and coevolution. But mitochondria do much more than make ATP; for example, they maintain redox and calcium balance, mediate apoptosis, regulate amino acid and lipid metabolism via the Krebs cycle and beta-oxidation, and influence TOR signaling, among many other functions [2]. So, there are additional nuclear genes that might influence mitonuclear coadaptation by indirect interactions with mtDNA-encoded functions.

Testing hypotheses of molecular coadaptation in the age of genomics is potentially rather easy. One can sequence the genomes or transcriptomes of different organisms and ask if the interacting genes or proteins show parallel patterns of molecular evolution. Because mtDNA genes usually evolve faster than nuclear genes, those that interact with mtDNA-encoded partners should show elevated rates of divergence, implying coevolution. For functional tests, genetic replacement of a foreign mtDNA in to the nuclear background of a different species should disrupt mitonuclear coadaptation and cause fitness defects. There are some nice cases in support of these molecular [11] and genetic predictions [12], but there are also some robust examples that have not uncovered the predicted patterns from coevolution [13–15]. So, why has putatively ongoing mitonuclear coevolution not left consistent footprints of selection that should be easy to discover? Are there systems that are more likely to reveal the expected patterns?

**Fig 1:**
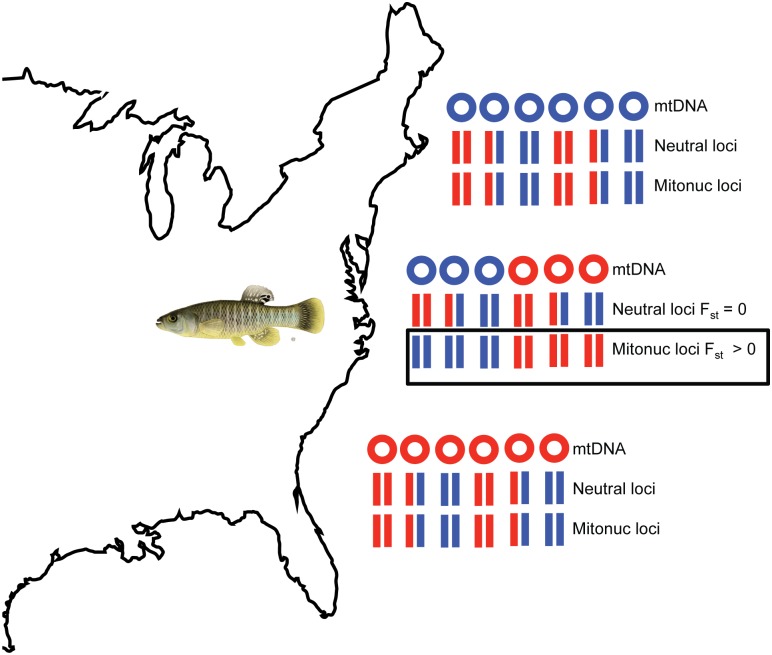
Nonrandom mitonuclear associations in a mixed population. In this simplified cartoon, the northern and southern populations of *F*. *heteroclitis* each have one mitochondrial DNA (mtDNA) haplotype (blue in the north, red in the south), and alleles at neutral nuclear loci and mitonuclear loci (Mitonuc loci) associated with mitochondrial function are in Hardy-Weinberg equilibrium ([HWE], for red and blue alleles). The New Jersey population has both mtDNAs in equal frequency, and the neutral loci show the same HWE frequencies in each mtDNA background, but the mitonuclear loci show significant differences in genotype frequencies in the two mtDNA haplotype backgrounds (F_st_ > 0, see boxed “Mitonuc loci”). The latter suggests a selective maintenance of mitonuclear interactions for a subset of nuclear loci that have epistatic fitness interactions with mtDNA haplotypes. Map image is from http://d-maps.com/carte.php?num_car=11867&lang=en. Fish image is from http://www2.dnr.cornell.edu/cek7/nyfish/Cyprinodontidae/mummichog.html.

In this issue of *PLOS Genetics*, Baris et al. [16] use the estuarine fish *Fundulus heteroclitis* to search for a signature of adaptive mitonuclear interactions, taking advantage of a natural experiment that is a geographic legacy of glacial retreat along the east coast of North America. They sampled a population of fish in Mantoloking, New Jersey, harboring two distinct mtDNA haplotypes that diverged during a period of geographic isolation imposed by the Hudson River basin that drained meltwater from the Wisconsin ice sheet. Following glacial retreat, “northern” and “southern” populations came in contact, and today, the New Jersey population has a ~50:50 ratio of two mtDNA haplotypes and a ~80:20 ratio of nuclear alleles typically found in Maine and Georgia. Baris et al. used genotyping by sequencing (GBS) to sample the nuclear genomes of fish from the New Jersey population and stratified the analysis of nuclear allelic variation by the mtDNA haplotype that each fish carried. The question they ask is whether there is evidence for population subdivision of nuclear alleles within a single population, associated with the divergent mtDNA haplotypes segregating in that population (Fig 1). Assuming neutral alleles and random mating, there should be no such mitonuclear association, especially because there is no physical linkage between nuclear chromosomes and cytoplasmic mtDNA.

Baris et al. found a few hundred single nucleotide polymorphisms (SNPs) spread across the nuclear chromosomes of *F*. *heteroclitis* that have significantly different allele frequencies in the two mtDNA “populations” within this single collecting locality (*cf*. 349 SNPs using *p* < 0.01 and 236 or 72 SNPs using a false discovery rate of 10% and 1%). Importantly, these SNPs show higher levels of allele frequency difference between mtDNA partitions within this population (measured as *F*_*st*_ “outliers”) than they do between the focal New Jersey population and geographically isolated populations in Maine and Georgia, a clear rejection of neutral expectations. Baris et al. quantified OXPHOS activity (ADP-dependent state-3 respiration) of mitochondria extracted from heart tissue from four mitonuclear genotypes that define pure parental and admixed hybrid individuals (e.g., mtDNA-Nuclear genotypes as: North-North, North-Mixed, South-Mixed, South-South) and show that these mitonuclear genotypes explain a significant portion of the variation in OXPHOS function.

It is tempting to invoke adaptive mitonuclear epistasis to explain these data, in which mtDNA haplotypes offer a sort of alternative metabolic niche for nuclear alleles within a population, akin to the classical Levene model of balancing selection via opposing selection in alternative habitats [17]. Baris et al. make a concerted effort to test alternative explanations for *F*_*st*_ outlier status of SNPs partitioned by mtDNA, by testing 1,000 alternative data partitions of >9,000 nuclear SNPs. None of these “nuclear-nuclear” *F*_*st*_ outlier tests uncovered as many outlier SNPs as the original mitonuclear outlier test, suggesting that the main result is not littered with many false positives.

One unexpected result from these analyses is that that none of the 349 *F*_*st*_ outlier loci map to known nuclear genes encoding subunits of the OXPHOS complexes. If this study really has identified loci important in mitonuclear coadaptation, it suggests that long-range *cis-* or *trans-*regulatory factors, or downstream pathway effects, are mediating the epistatic interactions between mtDNA haplotypes and nuclear loci other than those encoding subunits of OXPHOS complexes. Understanding the genetic basis of fitness variation in the wild is a central goal of ecological and evolutionary genetics, and the genes of central metabolism have long fascinated biologists as a logical place to search for the source of this variation [6]. Given the critical role that mitochondrial function plays in organismal performance, and the increasing knowledge of the diverse roles of mitonuclear communication in regulating homeostasis, there is a real hope that we can track down the biochemical bases of these kinds of nonneutral patterns in nature. The Baris et al. study offers a nice example of how admixed populations with divergent mtDNAs might serve as a natural genetic screen for the footprints of mitonuclear epistasis and coevolution that could point to unanticipated targets of selection on metabolic function.
